# Effect of a diabetes counseling programme on knowledge, attitude and practice among diabetic patients in Erode district of South India

**DOI:** 10.4103/0975-1483.76422

**Published:** 2011

**Authors:** R Malathy, MP Narmadha, S Ramesh, Jose M Alvin, Babu N Dinesh

**Affiliations:** *Department of Pharmacy Practice, Swamy Vivekanandha College of Pharmacy, Elayampalayam, Tiruchengode, Tamil Nadu, India*; 1 *Department of Diabetology, Kovai Medical Center, Erode, Tamil Nadu, India*; 2 *Department of Molecular Biology, Lab of Physiology, Yeungnam University, South Korea*

**Keywords:** Attitude, diabetes, knowledge, patient counseling, practices

## Abstract

The prevalence of diabetes in India has grown over the past decade. Diabetic patients develop complications due to poor awareness regarding the disease and inadequate glycemic control. Patient education is the most effective way to lessen the complications of diabetes and its management. A total of 207 (85 males and 122 females) type 2 diabetes mellitus patients were enrolled and randomized into test and control groups. Patients in the test group received counseling at each visit and information leaflets from the pharmacist; the control group patients received counseling and information leaflets only at the end of the study. A validated knowledge, attitude, and practice (KAP) questionnaire was administered to both test and control group patients at baseline and at final follow-up to assess awareness regarding disease management. Glucose and lipid levels were also evaluated at baseline and final follow-up in both the groups. At the end of the study, the KAP score of test group patients improved significantly (P<0.0001), whereas no significant changes were observed in control group patients. The postprandial blood glucose (PPBG) levels decreased significantly in the test group. Total cholesterol (TC), triglycerides (TGL), and low density lipoprotein levels (LDL) also showed a decrease in the test group. Thus, our study reveals that pharmacist counseling might be an important element in diabetes management programs.

## INTRODUCTION

Diabetes mellitus (DM) is a group of metabolic disorders characterized by hyperglycemia. It is associated with abnormalities in carbohydrate, fat, and protein metabolism, and results in chronic complications, including microvascular, macrovascular, and neuropathic disorders.[1] The prevalence of DM has risen dramatically over the past two decades. It is estimated that the prevalence of diabetes in adults worldwide will rise to 5.5% in 2025 (as compared to 4% in year 1995), with India contributing the major part.[2]

Many causes have been postulated for the rise in the number of cases, including urbanization, sedentary lifestyles, poor nutrition, and obesity. People with DM who wish to live normal lives l need to know a lot about their illness.[3] Thus, awareness on DM and its complication has become an integral and essential part of DM care for both health professionals and the patients themselves. Consequently, educational efforts to improve self-management are central components of any effective treatment plan. There is increasing amount of evidence that patient education is the most effective way to lessen the complications of diabetes and its management.[4]

Patient counseling is a process that improves patients‘ ability to cope with their disease and make informed decisions regarding management and medication. It helps motivate patients to change any harmful dietary and lifestyle habits.[5] Pharmacists are in a unique position to play a vital role in helping patients to cope with their disease.

There are virtually no epidemiological studies from Erode district of South India assessing the level of awareness of DM among the diabetic population. Hence, this research on disease awareness and the vital role of counseling in the management of DM. This study aims to assess the baseline levels of knowledge, attitude, and practices of diabetic patients visiting two multispecialty hospitals and one private diabetic clinic regarding disease management. The baseline results were used to develop a counseling program and to assess whether this intervention could produce any improvement in diabetes awareness and practices.

## MATERIALS AND METHODS

This study was conducted in two selected multispeciality hospitals and one diabetic clinic in Erode, Tamil Nadu, India, over a period of 9 months. Diabetic patients of both genders visiting any of these study sites, above 30 years of age and ready to sign the consent form, were included in the study. Pediatric patients and pregnant women were excluded from this study. The institutional research and ethics committee approved the study and issued a letter of permission to conduct the study.

Prior to starting any educational program it is appropriate to gauge the awareness level of the community under study by conducting a KAP study. This will help in implementing a health education program tailored to the needs of the particular community. A suitably designed and validated KAP questionnaire was administered at baseline and at the final follow-up to all the study patients to assess awareness regarding the disease and its management.[6] The questionnaire covered three areas: knowledge, attitude, and practice. There were a total of 25 questions, with 18 questions related to knowledge about diabetes, 4 questions to assess the attitude of the patient towards the disease, and 3 questions regarding practices (which reflect how the patients put their knowledge and attitude into action). This questionnaire was filled in at a face-to-face interview with the investigator. For scoring, 1 point was awarded for each correct answer and none for an incorrect or unsure answer; thus, the maximum possible score was 25.

At enrollment, the patient‘s demographic details, past and present medical and medication history, family history, body mass index (BMI; kg/m^2^), diet, and smoking status were obtained in a suitably designed patient profile form. Additionally, blood pressure and laboratory test values like fasting blood glucose, postprandial blood glucose, glycated hemoglobin (HbA_1_C), and lipid levels were recorded from the patient‘s file. The patients in the test group received counseling during each visit over a period of 3 months and the changes in the KAP score, glycemic control, and lipid profile were investigated.

Since there are no specific standards for diabetic counseling, the KAP questionnaire responses were analyzed and used to help develop an appropriate counseling programme. Pharmacist counseling sessions in the regional language were carried out for 20–25 min each visit at 1-month intervals over a period of 3 months. The pharmacist explained issues relevant to diabetes, such as pathophysiology and cause of diabetes, short- and long-term complications of diabetes, blood glucose control, recommendations for appropriate lifestyle changes (e.g., exercise, smoking cessation, etc.), nutrition recommendations, and foot care. After the first counseling session, the test group patients were provided printed handouts in the local language (Tamil) containing information on diabetes and desirable dietary and lifestyle changes. The patients in the control group received pharmacist counseling and patient information leaflets only at the end of the study.

### Definitions

*BMI:* The weight (in kilograms) divided by the square of the height (in meters). Classification of overweight and obesity were as per the recommendations of the National Heart, Lung, and Blood Institute, 1998.[7] According to this classification, patients with BMI of 18.5–24.9 kg/m^2^ were considered as normal, 25.0–29.9 kg/m^2^ was considered overweight, and 30.0-39.9 kg/m^2^ was considered obese.

*Smokers:* Individuals who reported to have smoked 100 cigarettes in their lifetime, irrespective of whether they were current smokers or not.

*Family history of DM:* “Diabetic patients whose first-degree relatives developed DM before the age of 60”? were categorized as patients with a positive history of DM.

Statistical analysis

KAP score and PPBG and lipid levels were compared using two-tailed unpaired *t* test. *P*<.05 was considered significant. The software used was GraphPad InStat 3.10.

## Results

A total of 207 patients fulfilling the inclusion and exclusion criteria were enrolled into the study and were randomized into the ‘test’ and ‘control’ groups. Of these patients, 85 (41.0%) were males and 122 (58.9%) were females. The average age of the test group subjects was 52.07 ± 9.47 and that of the control group subjects was 51.02 ± 9.83. The distribution of diabetic patients according to the sociodemographic characteristics is shown in Table 1.

**Table 1:** Sociodemographic characteristics of diabetic patients

Patients characteristics	Test group (*n*=137) Number (%)	Control group (*n*=70)Number (%)
Sex		
Male	39 (28.5)	46 (65.7)
Female	98 (71.5)	24 (34.3)
Age (years)		
<39	14 (10.2)	8 (11.4)
40–49	31 (22.6)	16 (22.9)
50–59	60 (43.8)	30 (42.8)
60–69	30 (21.9)	16 (22.9)
70 +	2 (1.5)	-
Educational level		
Illiterates	40 (29.2)	22 (31.4)
Primary school level and below	11 (0.8)	4 (5.7)
Up to secondary school	69 (50.4)	34 (48.6)
Graduates and above	17 (12.4)	10 (14.3)
DM history in family		
Present	35 (25.5)	30 (42.8)
Absent	102 (74.5)	40 (57.1)
BMI (kg/m^2^)		
Normal weight (18.5–24.9)	52 (38)	28 (40)
Overweight (25.0–29.9)	54 (39.4)	28 (40)
Obese (30.0–39.9)	31 (22.6)	14 (20)
Smoking status		
Yes	17 (12.4)	–
No	120 (87.6)	70 (100)
Duration of diabetes (years)		
<1	2 (1.5)	6 (8.6)
1–5	49 (35.8)	36 (51.4)
5–9	54 (39.4)	12 (17.1)
10 +	32 (23.3)	16 (22.9)

The educational status of the test group was assessed and showed that 40 (29.2%) subjects were illiterate, whereas 69 (50.4%) had been educated up to secondary school level. Only 35 (25.5%) of the test group was found to have a positive family history of DM, which shows the rapid emergence of DM among the general population. We also observed that 54 (39.42%) of the test population were overweight and 31 (22.6%) of them were obese, which indicates the poor level of awareness regarding the benefits of physical activity and exercises in reducing the BMI. Among the males 17 (12.4%) were smokers. In the test group, 21 (15.3%) patients had systolic BP ≥ 140 mm Hg and 36 (26.3%) had diastolic BP ≥ 90 mm Hg. Also, 54 (39.42%) patients in the test group had had diabetes for a period of 5–9 years.

We observed that in the test group, 70 (51.1%) patients were on treatment with oral hypoglycemic agents (OHA) and 41 (30%) were on diet control only. Diabetic complications (like history of decrease in weight, numbness in soles, inability to conceive, hypercholesterolemia, burning sensation in soles, swelling of legs, and fatigue) were found in both test and control groups. In the test group, fasting blood glucose level was >100 mg/dL (range: 106-302 mg/dL) in 94 (68.6%) patients. HDL level was in the range of 35–45 mg/dL in 74 (54%) patients as shown in Table 2.

**Table 2:** Biochemical and clinical characteristics of diabetic patients

	Test group (*n*=137) Number (%)	Control group (*n*=70) Number (%)
Blood pressure (mm Hg)		
Systolic		
<120	39 (28.5)	24 (34.3)
120–129	39 (28.5)	22 (31.5)
130–139	38 (27.7)	12 (17.1)
140 to+	21 (15.3)	12 (17.1)
Diastolic		
<80	40 (29.2)	26 (37.1)
80–89	61 (44.5)	26 (37.1)
90 to +	36 (26.3)	18 (25.8)
Latest fasting blood glucose level (mg/dl)		
≤100	43 (31.4)	24 (34.3)
> 100	94 (68.6)	46 (65.7)
High-density lipoprotein (mg/dl)		
<35	49 (35.8)	24 (34.3)
35 to < 45	74 (54)	36 (51.5)
45 to < 50	6 (4.4)	2 (2.8)
50 to < 60	6 (4.4)	4 (5.7)
> 60	2 (1.4)	4 (5.7)
Low-density lipoprotein (mg/dl)		
≤ 100	40 (29.2)	18 (25.7)
101–130	76 (55.5)	39 (55.6)
131–160	20 (14.6)	10 (14.4)
160	1 (0.7)	3 (4.3)
Triglycerides (mg/dl)		
≤ 200	114 (83.2)	62 (88.6)
201–240	16 (11.7)	6 (8.6)
241–280	5 (3.6)	2 (2.8)
> 281	2 (1.5)	-

The diabetes awareness level and the effect of pharmacist counseling were studied using a KAP questionnaire. On analyzing the responses, the percentage of patients in the test group who answered correctly were more at the final follow-up compared to the baseline. On evaluating the knowledge part of the questionnaire, we found that 46 (33%) patients were not aware of the consequences of high blood pressure. Among our respondents, only 69 (50%) knew that a common complication of diabetes was heart attack. Only 46 (33%) of patients knew about the importance of foot care. Another crucial finding of our study was that only 23 (16.7%) patients knew that urine examination could also be used to check glycemic levels. Also, only 23 (16.7%) of our respondents knew that the treatment of diabetes includes non-pharmacological measures such as lifestyle changes, regular exercise, and diet modification in addition to drug treatment [Table 3and 4]. Even though the overall KAP scores of the test group were significantly (P<0.0001) higher at the end of the study as shown in Table 4, the practice domain did not show any improvement (P<0.06), since the scores at the first visit itself was very high.

**Table 3:** Comparison of pre- and post-counseling knowledge scores as assessed by the KAP questionnaire

Questions	Test group Number of patients giving correct answers (%)		Control group Number of patients giving correct answers (%)	
	Before counseling	After counseling (After 3 months)	At baseline	After 3 months
Knowledge				
Diabetes is a condition in which the body contains…	103 (75)	126 (92)	48 (69)	48 (69)
The major cause of diabetes is…	69 (50)	114 (83)	36 (55)	37 (52)
The symptom (s) of diabetes is/are…	103 (75)	126 (92)	55 (75)	49 (70)
Diabetes, if not treated…	69 (50)	103 (75)	39 (56)	41 (59)
The most accurate method of monitoring diabetes is…	126 (92)	133 (97)	66 (95)	66 (95)
In a diabetic patient, high blood pressure can increase or worsen…	46 (33)	69 (50)	21 (30)	23 (33)
A diabetic patient should measure his or her blood pressure…	91 (67)	114 (83)	45 (65)	48 (69)
The lifestyle modification(s) required for diabetic patients is/are	114 (83)	137 (100)	59 (85)	59 (85)
A diabetic patient should have his or her eyes checked…	91 (67)	126 (92)	44 (63)	45 (65)
Regular urine tests will help in knowing…	23 (17)	57 (42)	13 (19)	13 (19)
The important factors that help in controlling blood sugar are…	80 (58)	103 (75)	38 (55)	37 (54)
A regular exercise regimen will help in …	69 (50)	114 (83)	37 (53)	41 (59)
The well-balanced diet includes…	80 (58)	91 (67)	36 (51)	35 (50)
For proper foot care, a diabetic patient…	46 (33)	91 (67)	25 (35)	25 (35)
Treatment of diabetes comprises…	114 (83)	126 (97)	56 (80)	57 (82)
Diabetes cannot be treated with…	23 (16)	57 (42)	13 (18)	13 (19)
Upon control of diabetes, the medicines…	80 (58)	126 (97)	42 (60)	46 (65)
How do you manage hypoglycemia symptoms…	34 (25)	91 (67)	18 (26)	17 (25)
Are you following a controlled and planned diet…?	69 (50)	91 (67)	34 (48)	34 (48)
Do you miss taking the doses of your diabetic medication…?	80 (58)	103 (75)	41 (58)	42 (59)
Are you aware of blood sugar levels falling below normal when you are taking drugs…?	57 (42)	80 (58)	28 (40)	28 (40)
Mean ± SD	74.61±29.2	103.71±24.02	37.80±14.63	38.28±35.97

**Table 4:** Comparison of pre- and post-counseling knowledge scores as assessed by the KAP questionnaire

Attitude	Test group Number of patients giving correct answers (%)		Control group Number of patients giving correct answers (%)	
	Before counseling	After xounseling (After 3 months)	At baseline	After 3 months
Do you exercise regularly…………..?	57 (42)	80 (53)	28 (40)	27 (39)
Are you following a controlled and planned diet……?	69 (50)	91 (67)	34 (48)	34 (48)
Do you miss taking the doses of your diabetic medication……?	80 (58)	103 (75)	41 (58)	42 (59)
Are you aware of blood sugar levels falling below normal when you are taking drugs……..?	57 (42)	80 (58)	28 (40)	28 (40)
Mean ± SD	65.75±11.05	88.5±10.96	32.75±6.18	32.75±6.89
Practice				
Do you check your blood pressure regularly?	137 (100)	137 (100)	70 (100)	70 (100)
Have you ever had your eyes examined?	137 (100)	137 (100)	70 (100)	70 (100)
Have you ever had your urine exam done?	137 (100)	137 (100)	70 (100)	70 (100)
Mean ± SD	137±0.0	137±0.0	70±0.0	70±0.0

**Figure 1:**
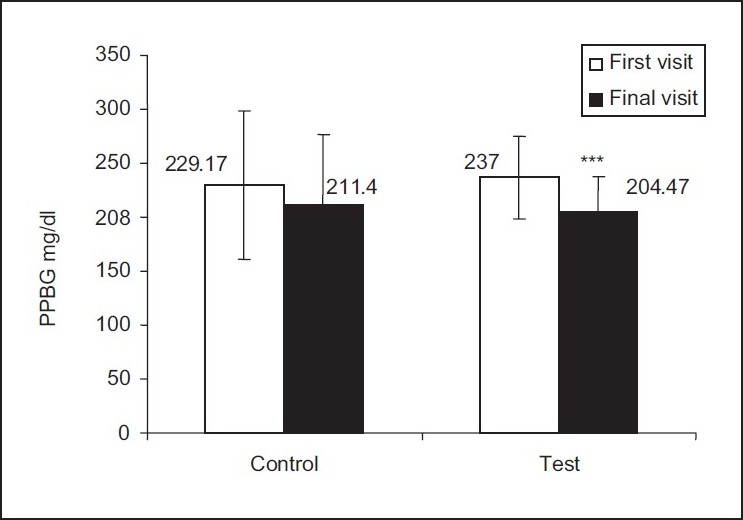
Comparison of PPBS levels between control and test groups Two-tailed unpaired t test was performed, ***P<.001

Although HbA _1_C (glycated hemoglobin) is the internationally accepted test, diabetologists in rural and semiurban areas generally advice their patients to go for postprandial blood glucose (PPBG) levels as the tool for monitoring glycemic control. The initial average PPBG level of the control group patients was 229.17 mg/dL and that of the test group patients was 237.0 mg/dL. The average PPBG levels of patients in the test and the control groups during the first and final visit are shown in Figure 1.

The percentage reductions in TC, LDL, and VLDL (very low density lipoprotein) did not differ much, while TGL levels reduced significantly from 150.9 mg/dL to 140.6 mg/dL (*P*<0.001) in the test group as compared to the control group where the reduction was from 155.7 mg/dL to 148.5 mg/dL. Similarly, HDL also increased significantly from 34.9 mg/dL to 36.6 mg/dL (*P*=0.05) in the test group [Table 8] as a result of pharmacist counseling.

**Table 8:** Impact of counseling on lipid profile

Lipid Profile		Test group	Control group
	I visit	206.2	202.8
TC (mg/dl)	After 3 months	185.7	191.5
	% Reduction	0.2***	0.1*
	I visit	150.9	155.7
TGL (mg/dl)	After 3 months	140.6	148.5
	% Reduction	0.1**	0.07
	I visit	34.9	33.4
HDL (mg/dl)	After 3 months	36.6	34.1
	% Increase	0.01*	0.007
	I visit	141.12	138.26
LDL (mg/dl)	After 3 months	120.9	127.2
	% Reduction	0.19***	0.1**
	I visit	5.9	6.07
TC / HDL	After 3 months	5.1	5.61
	% Reduction	0.008	0.004
	I visit	30.18	31.14
VLDL (mg/dl)	After 3 months	28.12	29.7
	% Reduction	0.02**	0.01**
			

Two-tailed unpaired t test was performed,

TC-Total cholesterol, TGL-triglycerides, HDL-high density lipoprotein, LDL-low density lipoprotein, VLDL-very low density lipoprotein

* *P*<.05

** *P*<.01

*** *P*<.001

## DISCUSSION

Diabetes has a negative impact on the patient‘s quality of life due to its many complications. Diabetic patients develop complications due to lack of awareness of the disease and inadequate glycemic control. There is increasing amount of evidence that patient education is the most effective way to lessen the complications of diabetes. [8]

A total of 207 patients were enrolled into this study. Male patients were more in number than females and the maximum number of patients were in the age-group of 50–59 years. This is similar to the study carried out in Western Nepal by Dinesh *et al*.[6]

About 65 (31.40%) of diabetic patients reported a positive family history of DM, which implies that more than one-fourth of the total study population had a genetic contribution to their diabetes etiology. It is astonishing to note that 142 (68.59%) diabetic patients had no family history of diabetes; this probably indicates the rapid emergence of diabetes among the general population. In India, the older members of the population who have had diabetes for a relatively long time are protected from risk of diabetic complications because of their physical activity patterns and dietary habits (making healthier food choices), while the current younger generation face high risk of diabetic complications due to a sedentary and stressful lifestyle. Over the past few years, the working patterns have changed, with fewer people involved in manual labor (e.g., as in the agriculture sector) and more and more people opting for physically less demanding office jobs. Another factor for the increase in risk for diabetes mellitus is the ‘fast food culture’ that has overwhelmed our cities and towns. The ‘fast foods’ that are rich in fats and calories are readily available in numerous food shops. As the majority of the young working population depend on these unhealthy ‘junk foods,’ this may partly explain the rise in diabetes incidence in the younger age-groups.

The question that arises is whether diabetic subjects are well-informed about their illness and its complications. Patient education constitutes a cornerstone in the management of diabetes. Knowledge regarding diabetes forms the basis for informed decisions about diet, exercise, weight control, blood glucose monitoring, use of medications, foot and eye care, and control of macrovascular risk factors.[9] Unless individuals with diabetes know that the disease can be transmitted to the offspring, steps cannot be taken to prevent diabetes in the high-risk group in the next generation. Several studies have reported the positive impact of counseling by clinical pharmacists on glycemic control and quality of life outcomes in the diabetic population.[10]

The higher percentage of correct answers from patients with positive family history of diabetes was not surprising. A study by Shobana *et al*.[11] showed that patients with positive family history were more aware of the role of heredity, of diet as a mode of therapy, and of the long-term complications of diabetes mellitus. Similarly, educational status improved knowledge regarding the disease. Paulose[12] carried out a disease awareness study in 400 literate diabetic patients in Kerala. The study found that although 80% of patients knew the symptoms of hypoglycemia and 76% knew what to do when they develop these symptoms, only 17% carried glucose packets with them during their travel; 29% patients said that their doctors had not informed them about possible hypoglycemic complications. These studies suggest that it is essential to educate the patients regarding proper diet, exercise, glucose control, and periodic consultations. Nilsson *et al*.[13] carried out a study to examine the relationship between educational level and mortality among diabetics. The study, conducted over a period of 16 years, enrolled 39055 subjects aged 25–75 years. The authors found that subjects who were less educated had a 40% excess cause of mortality compared with high educated subjects.

In the test group, 70 (51%) patients were taking oral hypoglycemic agents to control their diabetes, while 41 (30%) patients controlled diabetes with the help of diet alone [Table 5]. Therefore, it can be argued that pharmacist counseling may help patients to control blood sugar levels with diet changes alone and might help in postponing the use of OHA.

**Table 5:** Treatment details of diabetic patients

Treatment details	Test group (*n*=137) Number of patients (%)	Control group (*n*=70) Number of patients (%)
Type of treatment		
No treatment	7 (5.1)	4 (5.7)
Diet control	41 (30)	18 (25.7)
Oral hypoglycemic agents	70 (51.1)	36 (51.4)
(OHA)		
Insulin + OHA	3 (2.2)	1 (1.4)
Insulin + diet	1 (0.7)	1 (1.4)
OHA + diet	15 (11)	10 (14.4)
Diabetic complications		
Present		
Illiterates	35 (25.5)	29 (41.4)
Absent	102 (74.5)	41 (58.6)

The American Diabetic Association has advised that education on self-management is essential to provide the person with diabetes with the knowledge and skill that is needed to perform self-care, manage crises, and make lifestyle changes. The KAP questionnaire used in this study was developed to assess the perception of the patients about their disease and to assess the change in the perception after the pharmacist counseling.

The changes in the average KAP scores of each group (test and control) were statistically analyzed. The KAP score of test group patients improved significantly (P<.0001) after pharmacist counseling as compared to the control group [Table 6 and 7]. Previous research has shown that patient education adds value to diabetes management and that specific interventions aimed at improving patient knowledge can improve diabetes control.[14] It is well understood that diabetes management requires patient involvement for a better disease control. In our study, the patients lacked knowledge about the disease with a mean score of 9.8 ± 3.68 [Table 6]. Patients‘ knowledge levels can be improved in many ways, including through pharmacist counseling and conducting group awareness programs.

This study shows that pharmacist counseling helps to improve glycemic controlFigure 1. A significant reduction in the PPBG levels of the patients was observed in the test group (P<0.001), while no significant changes were observed in the control group. Many studies have shown that better glycemic control in type 2 diabetes mellitus is associated with fewer physical symptoms, better mood, and better sense of well-being, which ultimately leads to improved quality of life and improved economic benefits.[15,16] The landmark Diabetes Control and Complication Trials (DCCT-1996) and United Kingdom Prospective Diabetes Study (UKPDS) have conclusively demonstrated that glycemic control definitely affects the appearance and progression of the complications of chronic diabetes.

**Table 6:** KAP scores (test group)

Variables (*n*=137)	First visit	Final visit (After 3 months)	*P* value
Knowledge / 18	9.8 ± 3.68	12.92 ± 3.56	0.0001*
Attitude / 4	1.84 ± 0.88	2.76 ± 0.86	0.0001*
Practice / 3	2.80 ± 0.40	2.88 ± 0.32	0.06†
Overall / 25	14.53 ± 4.36	18.53 ± 4.26	0.0001*

Values are expressed as mean ± SD,

* - Highly significant

† - not significant

**Table 7:** KAP scores (control group)

Variables (*n*=70)	First visit	Final visit (After 3 months)	*P* value
Knowledge / 18	10.35 ± 6.22	10.29 ± 6.33	ns
Attitude / 4	1.94 ± 1.88	2 ± 1.83	ns
Practice / 3	3 ± 0	3 ± 0	ns
Overall / 25	15.3 ± 7.99	15.7 ± 8.06	ns

Values are expressed as mean ± SD,

^*^ - Highly significant; ns - not significant

This study also shows that pharmacist counseling helps to improve lipid levels [Table 8].

An encouraging part of this study is that many subjects were willing to wait patiently for their turn to attend the counseling session. This reflects the thirst for information among diabetic patients and also the lack of such programs in south India. A few patients were not quite comfortable with the pharmacist talking to them about their medications. These patients need to be convinced about the importance of this service and this can be done by establishing a trusting and professional relationship with them, which should motivate them to participate in such counseling programmes.

Pharmacists’ involvement in patient care has resulted in reduced number of hospital admissions and emergency department visits, as well as improved health status of patients.[17] There is considerable evidence that pharmacist-provided counseling enhances patient compliance and improves the quality of life outcomes in diabetes.[18] As evidenced from our results, the KAP score of the patients improved significantly (P<0.0001) after patient counseling by the pharmacist, with significant improvement in two of the three parameters of the analysis, viz., knowledge (P<0.0001) and attitude (P<0.0001).

## Conclusion

In India, the pharmacist is usually viewed as a person who merely fills the prescription and hands out the medications quickly. The observations of our study reveal a shift in the attitude of patients towards the pharmacist as a diabetic counselor. The results of the study also suggests that pharmacist counseling may have an impact in improving the perception about disease, diet, and lifestyle changes and thereby on glycemic control and the complications of diabetes. With the wealth of talented and skilled pharmacists in developing countries like India, we believe that such counseling programs if fine-tuned and implemented in diabetes management programs could definitely have immense impact on the profession of pharmacy, giving it an even greater place in the medical management of patients.
